# A Researcher’s Guide to the Measurement and Modeling of Puberty in the ABCD Study^®^ at Baseline

**DOI:** 10.3389/fendo.2021.608575

**Published:** 2021-05-05

**Authors:** Theresa W. Cheng, Lucía Magis-Weinberg, Victoria Guazzelli Williamson, Cecile D. Ladouceur, Sarah L. Whittle, Megan M. Herting, Kristina A. Uban, Michelle L. Byrne, Marjolein E. A. Barendse, Elizabeth A. Shirtcliff, Jennifer H. Pfeifer

**Affiliations:** ^1^ Developmental Social Neuroscience Laboratory, Department of Psychology, University of Oregon, Eugene, OR, United States; ^2^ Adolescent Research Collaborative, Institute of Human Development, University of California, Berkeley, Berkeley, CA, United States; ^3^ Cognitive-Affective Neuroscience and Development Laboratory, Department of Psychiatry, University of Pittsburgh, Pittsburgh, PA, United States; ^4^ Social Affective Neurodevelopment, Melbourne Neuropsychiatry Centre, Department of Psychiatry, The University of Melbourne and Melbourne Health, Melbourne, VIC, Australia; ^5^ Herting Laboratory, Department of Preventive Medicine, Keck School of Medicine, University of Southern California, Los Angeles, CA, United States; ^6^ Developing Brains Laboratory, Public Health & Institute for Interdisciplinary Salivary Bioscience Research, University of California, Irvine, CA, United States; ^7^ Turner Institute for Brain and Mental Health, School of Psychological Sciences, Monash University, Melbourne, VIC, Australia; ^8^ Stress Physiology Investigative Team, Human Development and Family Studies, Iowa State University, Ames, IA, United States

**Keywords:** adolescent brain cognitive development study, puberty, salivary hormones, testosterone, estradiol, DHEA

## Abstract

The Adolescent Brain Cognitive Development℠ (ABCD) Study is an ongoing, diverse, longitudinal, and multi-site study of 11,880 adolescents in the United States. The ABCD Study provides open access to data about pubertal development at a large scale, and this article is a researcher’s guide that both describes its pubertal variables and outlines recommendations for use. These considerations are contextualized with reference to cross-sectional empirical analyses of pubertal measures within the baseline ABCD dataset by Herting, Uban, and colleagues (2021). We discuss strategies to capitalize on strengths, mitigate weaknesses, and appropriately interpret study limitations for researchers using pubertal variables within the ABCD dataset, with the aim of building toward a robust science of adolescent development.

## Introduction

Pubertal measures provide critical information about maturation beyond chronological age, and there is substantial variation in the timing of pubertal milestones (1, 2). The Adolescent Brain Cognitive Development (ABCD) Study measures puberty across adolescence in a large and diverse sample (baseline ages = 9–10 years; annual sampling of pubertal measures for 10 years (ongoing); 21 research sites across the United States; N = 11,880; 48% female at baseline (3); see (4) for recruitment details). This represents an unprecedented opportunity to better understand relationships between puberty, sociodemographic variables, neurodevelopment, and health. The purpose of this article is to support scientists in planning and interpreting research about puberty from this dataset, particularly in light of what is known from recent analyses of the baseline data by Herting, Uban, and colleagues (5). We—a collaboration of puberty researchers including authors within and external to the ABCD consortium—seek to provide a balanced presentation of the study’s strengths, limitations, opportunities (areas of growth/innovation), and threats (issues endangering the validity of potential interpretations) as they pertain to puberty. We further explore practical considerations for planning investigations with these data, including a brief discussion of longitudinal analyses.

## ABCD Study Design: Implications For Pubertal Research

This section addresses aspects of the study design that are relevant to studying puberty, including sample composition and assessment frequency [for information about specific measures in the ABCD Study, see the section on *Pubertal measures in the ABCD dataset*; for general recommendations regarding pubertal measurement/modeling, see (6, 7)]. Puberty is comprised of distinct (albeit temporally overlapping) hormonal processes that drive specific physical changes. Adrenarche involves a rise in adrenal hormones [testosterone (T); dehydroepiandrosterone (DHEA) and its sulfate], while gonadarche involves a rise in gonadal hormones [estradiol (E2) and progesterone from the ovaries; T from the testes; for a review, see (8)]. As the suffix -*arche* refers to first occurrences, we use the terms adrenal and gonadal processes to refer to the multi-year maturation of each endocrine axis (hypothalamic–pituitary–adrenal and hypothalamic–pituitary–gonadal). The maturation of the growth (hypothalamic–pituitary–somatic) axis is associated with increases in growth hormone and regulation of overall growth and metabolism (9).

### Strengths

The large sample size and narrow age range facilitate well-powered investigations related to pubertal development that can be contextualized using sociodemographic variables (5) and will likely provide a basis for normative pubertal development in the USA population at the beginning of the 21st century. By following participants over a decade, growth curves will describe average growth and typical variation across adolescence. Analyses of the baseline data suggest that there is sufficient variance to relate individual differences in pubertal development to other measures (5). Furthermore, the field of pubertal research has largely focused on girls of European descent, and examination of puberty in males and in racially/ethnically diverse participants fills in critical knowledge gaps (10, 11).

### Limitations

Due to participant ages at recruitment, the study is unable to capture many adrenal processes for nearly all participants and early gonadal processes for many participants―especially females (5). This limitation is more pronounced for groups that start puberty earlier relative to the average, including Black and non-White Hispanic participants (12, 13), those from lower levels of socioeconomic status (14), and those with higher BMI (15) [trends replicated in the ABCD data at baseline (5)]. Puberty is classically considered complete following development of secondary sexual characteristics and capability for reproduction (roughly mid-adolescence; later for boys relative to girls). This prevailing definition is complicated by the fact that hormone levels (16) and body composition (17) mature into the late teens and 20s. The study plans to follow participants to 19–20 years of age, but this would fall short of addressing questions about how late hormonal changes may affect processes like risky decision-making at the ages when binge drinking (18) and other health risk behaviors peak (19). Additionally, pubertal development is non-linear with meaningful changes occurring at sub-annual time scales (*e.g.*, growth spurts) that are not captured (20).

## Pubertal Measures In The ABCD Dataset

Puberty-related variables in the ABCD Study are listed in **Table 1**. For descriptive statistics of these variables in the baseline data, see (5).

**Table 1:** Summary of puberty variables within the ABCD Study.

Content	Description	Sex*	Informant/Source	NDAR Element Name (Alias)**
**Pubertal Development Scale and Menstrual Cycle Survey History (Youth: abcd_ypdms01, Caregiver: abcd_ppdms01)**				
Sex/gender (not disambiguated in the question wording)	Do you consider yourself male or female?	All	Youth	pds_sex_y (pubertdev_sex)
Sex at birth	What sex was your child assigned at birth?	All	Caregiver	pubertal_sex_p (pubertdev_sex_p)
Height spurt	Would you say that your/your child’s growth in height 1 = has not yet begun to spurt; 2 = has barely started; 3 = is definitely underway; 4 = seems complete; 999 = I don’t know; 777= refuse to answer	All	Both	pds_ht2_y, pds_ht2_p (pubertdev_ht, pubertdev_1_p)
Skin changes	Have you/your child noticed any skin changes, especially pimples? 1 = skin has not yet started changing; 2 = skin has barely started changing; 3 = skin changes are definitely underway; 4 = skin changes seem complete; 999 = I don’t know; 777 = refuse to answer	All	Both	pds_skin2_y, pds_3_p*** (pubertdev_skin, pubertdev_3_p)
Body hair	And how about the growth of your/your child’s body hair? 1 = has not yet begun to spurt; 2 = has barely started; 3 = is definitely underway; 4 = seems complete; 999 = I don’t know; 777 = refuse to answer	All	Both	pds_bdy_hair_y, pds_2_p*** (pubertdev_bdyhair, pubertdev_2_p)
Breast development	Have you noticed that your/your child’s breasts have begun to grow? 1 = have not yet started growing; 2 = have barely started growing; 3 = breast growth is definitely underway; 4 = breast growth seems complete; 999 = I don’t know; 777 = refuse to answer	Female	Both	pds_f4_2_y, pds_f4_2_p (pubertdev_f4, pubertdev_f4_p)
Menstruation began?	Have you/she begun to menstruate (started to have your period)?	Female	Both	pds_f5_y, pds_f5_p (pubertdev_f5, pubertdev_f5_p)
Age of first period	If yes, how old were you/she when you/she started to menstruate?	Female	Both	pds_f6_y, pds_f6_p (pubertdev_f6, pubertdev_f6_p)
Deepening voice	Have you noticed a deepening of your/your child’s voice?	Male^1^	Both	pds_m4_y, pds_m4_p (pubertdev_m4, pubertdev_m4_p)
Facial hair	Have you/your child begun to grow hair on your/his face?	Male^^1^^	Both	pds_m5_y, pds_m5_p (pubertdev_m5, pubertdev_m5_p)
First day of last period	What was the date of the first day of your/your daughter’s last period?	Female	Both	menstrualcycle1_y, menstrualcycle1_p (menstrual_1, menstrual_1_p)
Cycle length	On average, how many days are there between the first day of your/her period and the first day of your/her next period? (e.g., 30 days)	Female	Both	menstrualcycle2_y, menstrualcycle2_p (menstrual_2, menstrual_2_p)
Cycle regularity	Is your/her menstrual cycle regular?	Female	Both	mentrualcycle3_y, menstrualcycle3_p (menstrual_3, menstrual_3_p)
Hormonal birth control	Are you/is she currently using hormonal birth control (eg. the pill, hormone patch, hormone injection)?	Female	Both	menstrualcycle4_y, menstrualcycle4_p (menstrual_4, menstrual_4_p)
Premenstrual irritability	Do you/does she experience premenstrual symptoms, such as irritability, fatigue, etc., which start before a period and stop within a few days of bleeding?	Female	Both	menstrualcycle5_y, menstrualcycle5_p (menstrual_5, menstrual_5_p)
Does PMS interfere with activities	Do your/her premenstrual symptoms interfere with your/her relationships with family and friends, productivity, and/or social life activities?	Female	Both	menstrualcycle6_y, menstrualcycle6_p (menstrual_6, menstrual_6_p)
**Derived pubertal development scores (ABCD Sum Scores Physical Health Youth and Parent; abcd_ssphp01)**				
Category score based on the Pubertal Development Scale (prepubertal/early/mid/late/post)^3^		All	Both	pds_y_ss_female_category (pubertdev_ss_female_category), pds_y_ss_male_category (pubertdev_ss_male_category), pds_p_ss_female_category (pubertdev_ss_female_category_p), pds_p_ss_male_category (pubertdev_ss_male_category_p)
**Salivary hormone measures (Hormone Saliva Salimetric Scores; hsss01)**				
Salimetrics hormone test mean (pg/ml)	Hormone saliva variable recorded by Salimetrics	dhea: all, hse (E2), females only; ert (T): all	Youth	hormone_scr_dhea_mean, hormone_scr_hse_mean, hormone_scr_ert_mean
Salimetrics hormone test below lower limit of sensitivity^2^, replications 1 and 2**** (yes/no)				hormone_scr_dhea_rep1, hormone_scr_hse_rep1, hormone_scr_ert_rep1
Salimetrics hormone quantity not sufficient, repetitions 1 and 2**** (yes/no)				hormone_scr_dhea_rep1_qns, hormone_scr_hse_rep1_qns, hormone_scr_ert_rep1_qns
Salimetrics hormone test none detected, repetitions 1 and 2**** (yes/no)				hormone_scr_dhea_rep1_nd, hormone_scr_hse_rep1_nd, hormone_scr_ert_rep1_nd
**Other variables**				
Sex (male/female)		All	Caregiver	sex (sex_at_birth, also aliased as “gender”)
Mean standing height (inches)		All	Youth	anthroheightcalc (anthro_height_calc [mean of anthro_1_height_in, anthro_2_height_in, anthro_3_height_in])
Mean weight (lbs)		All	Youth	anthroweightcalc (anthro_weight_calc [mean of anthro_weight1_lb, anthro_weight2_lb, anthro_weight3_lb])
**Quality control measures for saliva samples (Pubertal Hormone Saliva information at time of fluid collection; sph01)**				
Wake-up time on test day	What time did you wake up today?	All	Youth	hormone_sal_wake_y (biospec_hormone_sal_wake)
Caffeine	Have you had any caffeine in the last 12 hours? How many drinks (mg) did you have	All	Youth	hormone_sal_caff_y (biospec_hormone_sal_caff)
				hormone_sal_caff_mg_y (biospec_hormone_sal_caff_mg)
Exercise	In the last 12 hours, did you exercise vigorously (sweating, breathing hard) for at least 20 minutes? For how long did you exercise?	All	Youth	hormone_sal_active (biospec_hormone_sal_active)
				hormone_sal_active_minutes_y (biospec_hormone_sal_active_minutes)
Sample Collection information	Hormone saliva tube cap color, Sample time collection start, Sample time collection end, Sample barcode, Storage temperature, Time sample moved to freezer	All	Youth	hormone_sal_sex, hormone_sal_start_y, (biospec_hormone_sal_start), hormone_sal_end_y, (biospec_hormone_sal_end) hormone_sal_bc_y, (biospec_hormone_sal_bc) hormone_sal_freeztemp_y, (biospec_hormons_sal_freeztemp) hormone_sal_freezer_y (biospec_hormone_sal_freezer)
Concerns about sample	None, Contaminated, Discoloration, Excessive bubbles, Insufficient quantity, Other	All	Youth	hormon_sal_notes_y:_1 through hormon_sal_notes_y:_6 biospec_hormon_sal_notes___1 through biospec_hormon_sal_notes___6)

Adapted from ABCD data release 2.0.1, last updated 05/06/2020 (doi: 10.15154/1506087; wave 01/baseline). The ABCD data repository grows and changes over time. The contents under NDAR Element Name (Alias) refer to abbreviations in the ABCD dataset codebook, and may be useful only to those who have successfully applied for (free) access to the data. *Sex-specific scores were calculated based on responses to the sex_at_birth variable, which only had binary response options; we note that there are various sex and gender related variables, and that a separate scale was administered to address gender identity (not described above); ** Throughout, “y”; denotes youth and “p”; denotes parent/caregiver versions; *** Number mismatch is because youth and caregiver measures were in a different order; **** Due to space constraints, only abbreviations for repetition 1 are shown. ^1^Only boys responded to these questions. This limits the documentation of girls with androgen excess who may also experience facial hair and deepening of the voice. ^2^The lower limit of sensitivity is reported for each hormone in Herting, Uban, and colleagues (2021). ^3^Converted Pubertal Development Scale values are not provided and must be calculated by researchers [see (5) for more information on possible calculations].

### Measures of Physical Maturation

#### Strengths

The study assesses physical development using the Pubertal Development Scale (PDS), a minimally invasive, text-only measure designed for ease of administration (21). On the PDS, individuals rate their own/their child’s development on a four-point Likert scale from “had not begun” to “already complete” with respect to specific physical characteristics (*e.g.*, skin changes, breast development; a subset of the items was administered based on sex). The PDS is versatile, as researchers might use the mean PDS score, converted scores made to be more comparable to Tanner staging (22, 23), derived scores intended to reflect adrenal and gonadal processes separately, and/or focus on a specific item (*e.g.*, age at menarche). Collecting data from multiple informants allows researchers to use caregiver reports at earlier ages and adolescent reports at later ages [for examples, see (24, 25)]. Prioritizing caregiver reports may be useful at baseline considering the large number of “I don’t know” responses to several items (notably 34–42% for growth spurts; 10% for menstruation) (5); at these ages, caregivers may have greater knowledge of where adolescents are in the process of change. Adolescent reports may better reflect intimate experiences with body changes over time, particularly at later ages (26). Adolescent self-report may be an ideal measure for studies focused on the consequences of puberty for social- or self-related processes (21), and is sometimes as closely or more closely associated with hormone levels than Tanner staging *via* clinical examination (27, 28).

#### Limitations

When using the PDS and its derived scales, consider the following limitations: First, the PDS does not evaluate pubertal stage directly, and a description of the construct is better reported as “perceived pubertal stage,” although it is uncommon to do so (29). While there are conversions transforming the PDS to values more comparable to Tanner staging, some recommend that the PDS should not be used when precise Tanner staging is of interest (20). Second, the PDS does not cover the full range of puberty equally well (solicits less information about earlier changes) (29). Third, the PDS exhibits systematic discrepancies with clinician ratings. Consistent with desirability effects, relatively less advanced adolescents tend to overestimate their PDS score, while more advanced adolescents tend to underestimate (27, 30).

### Pubertal Hormone Measures

#### Strengths

The study provides objective measures of the quantity of biologically available DHEA, T, and E2 [in girls only; for details on hormone methods see (5)] at remarkable scale *via* salivary measures. From gonadarche onwards, increases in DHEA and T in males reflect adrenal and gonadal processes, respectively. In females, DHEA and T largely reflect adrenal processes, while E2 levels reflect gonadal ones. Hormone levels are not redundant with information about physical maturation. In the ABCD data at baseline, PDS summary scores were modestly correlated with hormone levels [0.12–0.20 in males; 0.10–0.34 in females (5)]. In another study of 9–14 year-olds, PDS-derived scores accounted for 35–40% of the variance in DHEA and T levels in males and 15–27% of DHEA, T, and E2 levels in females (27). Associations between circulating hormone levels and physical changes vary by factors including sex, race/ethnicity, and body mass index (5, 15, 31). Examining hormone levels may be particularly useful during the earliest and latest pubertal stages: Adrenal hormone levels rise prior to physical changes associated with adrenarche (32), and circulating levels of DHEA, T, and E2 rise after the ages at which adolescents reach Tanner stage V (sometimes considered the last pubertal stage) (16).

Data for potential hormone quality confounds were recorded at the time of sampling, including time of day, caffeine consumption, and medication use (for a list, see **Table 1**); linear effects on hormone levels have been estimated at baseline (5). We encourage researchers to use transparent and reproducible processing procedures (*i.e.*, following a pre-specified decision-tree as implemented in publicly available scripts by Herting, Uban, and colleagues (5); https://figshare.com/articles/software/R_scripts/12673754).

#### Limitations

Due to feasibility issues, saliva was sampled once per visit and time of day varied widely [7 am–7 pm (5);]. Hormone levels fluctuate dynamically and non-linearly over various time-scales, and reliance on a single biospecimen renders researchers unable to account for momentary, daily/diurnal, or monthly hormonal fluctuations. Time of day is a major source of variability; early in puberty, each of these hormones exhibit non-linear diurnal rhythms with peaks in the morning, and these diurnal patterns further vary across pubertal development (33–35). Another major source of complex unmeasured variability is menstrual cyclicity in females: Even prior to menarche, cyclic changes in hormone levels can be detected, and variability in the menstrual cycle persists almost 2 years following menarche (7), with diurnal E2 rhythms attenuated approximately a year after menarche (35). Including time of day as a linear covariate may not sufficiently account for such effects. Sensitivity analyses within participants sampled during a restricted time window (and that reflect a random subsample across sites and demographics) may improve the validity of investigations employing these measures. Additionally, estimates from salivary assays are known to be less reliable at the extremes. We further note that other pubertal hormones were not assessed (notably progesterone, luteinizing hormone, and follicle stimulating hormone).

## Discussion

### Opportunities to Advance Our Understanding of Puberty

The ABCD Study presents the opportunity to parse the relative contributions of puberty and sociodemographic variables to adolescent development. Another opportunity, arising from the narrow age range and large sample size, is to disentangle effects of puberty and age (also see the section on *Considerations for Building Models Incorporating Puberty*). The study may also advance the development of multimethod pubertal measurement approaches. Herting, Uban, and colleagues (5) implemented group factor analyses with the ABCD baseline data to identify latent pubertal factors while accounting for method-related variance. They found a two-factor structure accounting for a combined 47.4% of the variability in pubertal measures in females and 38.6% in males. Researchers have typically focused on physical maturation or hormone levels separately, and novel multimethod approaches may approaches may contribute to longstanding questions in the field. (However, combining methods may not be necessary, and single-method approaches may be preferable for targeted research questions—for more on variable selection, see the section on *Strategizing for open and reproducible analyses*). For example, one question is the extent to which obesity itself is directly linked to early pubertal development rather than systematic measurement error, particularly overestimation of breast development in girls (15, 36). Group factor analyses with the ABCD baseline data found that greater body mass index was associated with higher-than-average hormone and physical maturation levels (higher Factor 1 scores), as well as more advanced physical maturation relative to hormone levels, compared to the sample average (higher Factor 2 scores). Advancement along both of these axes, relative to overall sample, is consistent with earlier pubertal development and suggests that measurement error of physical characteristics may not fully account for associations between obesity and more advanced puberty.

### Threats Endangering the Validity of Potential Interpretations

Conclusions drawn from the ABCD Study will undoubtedly carry impact. However, we should be cautious when developing major conclusions regarding certain aspects of puberty. The lack of pre-pubertal female participants is more pronounced in groups found to start puberty earlier (5). These sociodemographic differences must be carefully considered in work addressing pubertal onset and/or the very earliest stages of puberty. Early pubertal onset and timing contribute to risk for health problems (37, 38), and this issue may bias estimates of pubertal timing-related effects. Its extent will remain unknown until scientists and funding agencies invest widely in puberty-related research at earlier ages, particularly for Black and non-White Hispanic girls.

To mitigate general design and measurement limitations and to guard against misinterpretation, we recommend the following minimum standards for puberty research using this dataset: (a) When using physical maturation data, language reflects that the PDS is a measure of self- or parent-perceived pubertal maturation, (b) when using salivary hormone data, data are processed using a standardized and/or publicly available analysis pipeline and sensitivity analyses consider the effect of time of day on hormone levels, and (c) exude caution when drawing conclusions regarding precise pubertal stages and hormone levels, in part by acknowledging measurement limitations (see *Pubertal Measures in the ABCD Dataset for details on strengths and limitations*).

### Strategizing for Open and Reproducible Analyses

Like other complex constructs, puberty can be operationalized and modeled in ways that impact conclusions about its effects. For example, conclusions as to whether early pubertal maturation affects adult height in females differ when maturation is defined in terms of menarche *versus* breast development (39). This enhanced analytic flexibility (40) can have untoward effects in null-hypothesis significance testing, some of which can be mitigated by preregistration (41). While not comprehensive, potential *a priori* justifications for variable selection are presented in the section on *PUBERTAL MEASURES in the ABCD DATASET*. Decisions might also be informed by psychoneuroendocrinology, and neuroimaging analyses might consider what is known about hormone receptor types and density in brain tissue (42). Otherwise, Specification Curve Analysis, also known as multiverse analysis, can facilitate reporting of results from multiple model specifications that are consistent with the underlying theory; this approach can estimate the robustness of effects across numerous operationalizations of pubertal development (43–45) [for an example using pubertal variables, see (46)]. Pre-specifying the smallest effect size of interest (47) and/or employing the use of discovery samples for model-fitting and replication or holdout samples for model testing might also be useful (48), especially because small effects are likely to be significant when sample sizes are large.

### Considerations for Building Models Incorporating Puberty

There is no ideal or standard modeling approach, as decisions should reflect the research questions at hand. Drawing from our own work, we provide a few examples of possible approaches and highlight relevant considerations.

In one study, Ladouceur and colleagues (49) used measures of physical maturation (standardized composite scores) to separately consider effects of adrenal and gonadal processes. These scores, as well as hormone levels, were used as predictors in separate multiple linear regression models examining effects of puberty (controlling for age) on neural indices of reward processing in 10 to 13 year-olds (N = 79). This approach separately considered adrenal and gonadal processes, as well as physical and hormone measures, in order to disentangle various effects. Another study by Whittle and colleagues (50) showcased the use of exploratory factor analyses across multiple age-adjusted questionnaire items (including parent and child report PDS) to create a standardized pubertal timing measure (calculated at approximately age 12; N = 155). This measure was used in longitudinal analyses examining associations with pituitary volume and depressive symptoms. Longitudinal work by Vijayakumar et al. (51) compared nested mixed effects models (ages 9–18; N = 82) that predicted signal from a functional neuroimaging task from linear and quadratic effects of maturation (age and self-reported PDS scores converted into a Tanner-like scale). Analyses used both hormone and questionnaire measures by examining the impact of including T in best-fitting PDS models.

We recommend that researchers account for age when examining puberty (despite the narrow age window) because age was associated with each pubertal measure in the baseline data (6). Each of these studies highlights a different approach to disentangling puberty and age effects, specifically by including age as a covariate (49), creating age-adjusted pubertal indices (50), or by comparing age and puberty models (51).

Selecting and discretizing sex and gender variables requires careful theoretical and ethical consideration. Researchers should take care to use the terms sex and gender appropriately (52), to consider that they operate on developmental outcomes *via* different mechanisms (53), and to avoid essentializing either one as strictly binary (54). Youth and caregivers report on gender identity (55), but information about sex chromosomes or endocrine disorders is not formally acquired. In analyses highlighted above, researchers used composite or factor scores computed within sex, included sex as a covariate, and tested for interactions between sex and conditions of interest (49, 50). When evaluating hormones as putative sex-specific mechanisms, authors ran separate models by sex (49, 51).

Finally, physical maturation as measured by the PDS is ordinal, but many analyses (including those highlighted above) treats it as continuous; this specification is flawed because it implies that differences between all pubertal stages are equally meaningful or spaced with respect to some outcome. Some developmental shifts, *e.g.*, in human face perception, are associated with transitions between certain pubertal stages only (56).

### Opportunities and Challenges for Longitudinal Analyses

As more waves of ABCD data are released, there will be opportunities to describe sample norms and normed relative categorizations (*e.g.*, describing timing as early, typical, or late) with accompanying hormone trajectories. Longitudinal data will also allow for new variables such as age at menarche or peak height velocity (7) and modeling techniques such as non-linear latent growth modeling (57). Challenges include measurement invariance, as pubertal measures may change in their accuracy (*e.g.*, parent *versus* child reports) and/or substantive meaning (*e.g.*, E2 reflecting diurnal *versus* monthly patterns). Another documented phenomenon is that some adolescents will regress on pubertal maturation as measured by the PDS (21). While commonly associated with measurement error and the coarseness of the measure (21), further investigation of regression within the ABCD Study is warranted, including consideration that adolescents’ self-perceptions may change over time. Finally, as the number of self-identified sexual and gender minority youth increase longitudinally (55) there will be opportunities to study puberty in gender diverse youth, and these groups should be consulted when conducting and interpreting this research.

### Conclusion

This article outlines critical considerations for investigations using pubertal data from the ABCD Study. Major strengths include the size and demographic diversity of the sample and the use of both questionnaires and salivary hormones. Limitations include the inability to investigate earlier aspects of puberty and an inability to account for multiscale hormone fluctuations, such that smaller longitudinal studies are still needed to answer open questions about puberty. Overall, we recommended that researchers using these data describe the PDS as perceived rather than objective pubertal development, use sensitivity analyses to account for diurnal hormone cycles, prioritize open and reproducible science, and account for age in their models despite the narrow age band. We hope that facilitating a better understanding of the ABCD Study’s design and measures will ultimately support a stronger science of adolescent development.

## Data Availability Statement

Publicly available datasets were analyzed in this study. This data can be found here: Researchers can apply for access to the data at https://nda.nih.gov/abcd/request-access.

## Author Contributions

Authors TC, VGW, LM-W, and CL wrote different portions of this manuscript. All authors participated in extensive feedback, comments, and discussion, including TC, VGW, LM-W, CL, MEAB, SW, MLB, ES, MH, KU, and JP. All authors contributed to the article and approved the submitted version.

## Funding

This project was conceptualized at the ABCD Workshop 2019, which was supported by the National Institute of Mental Health of the National Institutes of Health under Award Number R25MH120869. Author TC was supported by the National Center for Advancing Translational Sciences of the National Institutes of Health under award number TL1TR002371. Author CL was supported by the National Institute of Mental Health of the National Institutes of Health under Award Number MH099007. Author MLB was supported by the National Institute of Mental Health of the National Institutes of Health under Award Number K01MH111951. Author MH was supported by the National Institute of Mental Health under Award Number: K01 MH10876. Author SW was supported by the National Health and Medical Research Council under award number 1125504. Author KU was supported by the National Institute on Alcohol Abuse and Alcoholism under Award Number: K01 AA026889. Author JP was supported by the National Institute of Mental Health under award number MH174108. To prepare this article, we examine and present details about measures administered in the Adolescent Brain Cognitive Development (ABCD) Study (https://abcdstudy.org), held in the NIMH Data Archive (NDA). This is a multi-site, longitudinal study designed to recruit more than 10,000 children ages 9–10 and follow them over 10 years into early adulthood. The ABCD Study is supported by the National Institutes of Health and additional federal partners under award numbers U01DA041048, U01DA050989, U01DA051016, U01DA041022, U01DA051018, U01DA051037, U01DA050987, U01DA041174, U01DA041106, U01DA041117, U01DA041028, U01DA041134, U01DA050988, U01DA051039, U01DA041156, U01DA041025, U01DA041120, U01DA051038, U01DA041148, U01DA041093, U01DA041089. A full list of supporters is available at https://abcdstudy.org/federal-partners.html. A listing of participating sites and a complete listing of the study investigators can be found at https://abcdstudy.org/scientists/workgroups/. ABCD consortium investigators designed and implemented the study and/or provided data but did not necessarily participate in analysis or writing of this report. The content is solely the responsibility of the authors and does not necessarily represent the official views of the NIH or ABCD consortium investigators. Author TC was supported by the National Center for Advancing Translational Sciences of the National Institutes of Health under award number TL1TR002371 and by the National Institute of Mental Health under award number 1F31MH124353.

## Conflict of Interest

The authors declare that the research was conducted in the absence of any commercial or financial relationships that could be construed as a potential conflict of interest.

## Footnotes

1 Only boys responded to these questions. This limits the documentation of girls with androgen excess who may also experience facial hair and deepening of the voice.

2 The lower limit of sensitivity is reported for each hormone in Herting, Uban, and colleagues (2021)
